# Effects of immersive virtual reality stimulation and/or multicomponent physical exercise on cognitive and functional performance in hospitalized older patients with severe functional dependency: study protocol for a randomized clinical trial

**DOI:** 10.1186/s12877-024-05516-x

**Published:** 2024-11-08

**Authors:** Antón de la Casa-Marín, Fabiola Zambom-Ferraresi, Maria Cristina Ferrara, Iranzu Ollo-Martínez, Arkaitz Galbete, Belén González-Glaría, Débora Moral-Cuesta, Itxaso Marín-Epelde, Chenhui Chenhuichen, Marta Lorente-Escudero, Rodrigo Molero-de-Ávila, Agurne García Baztán, Fabricio Zambom-Ferraresi, Nicolás Martínez-Velilla

**Affiliations:** 1 Department of Geriatric Medicine - Hospital Universitario de Navarra (HUN), Navarrabiomed, Universidad Pública de Navarra (UPNA), Instituto de Investigación Sanitaria de Navarra (IdiSNA), Pamplona, Spain; 2 School of Medicine and Surgery, University of Milan-Bicocca, Milan, Italy; 3 Department of Statistics, Computer Science and Mathematics, Universidad Pública de Navarra (UPNA), Pamplona, Spain; 4 CIBER of Frailty and Healthy Aging (CIBERFES), Instituto de Salud Carlos III, Madrid, Spain; 5 Clinical Research Department, TDN, Orthopedic Surgery and Advanced Rehabilitation Center, Mutilva, Spain; 6 School of Medicine, University of Navarra, Pamplona, Spain; 7 Hospital Universitario de Navarra, Irunlarrea 3, Pamplona, 31008 Spain

**Keywords:** Hospital-associated functional decline, Cognitive stimulation, Virtual reality, Multicomponent physical exercise, Acute care, Older adults

## Abstract

**Background:**

Hospital-associated functional decline affects nearly one-third of the hospitalized older adults. The aim of this trial is to investigate the effect of a cognitive stimulation intervention provided via immersive virtual reality (IVR), with or without a multicomponent physical exercise intervention (ME) in hospitalized patients aged 75 or older with severe functional dependency at admission (Barthel Index < 60 points).

**Methods:**

This clinical randomized controlled trial will be conducted in the Acute Geriatric Unit of a tertiary hospital in Spain. A total of 212 acute patients will be enrolled according to the following criteria: age *≥* 75, Barthel Index < 60, able to collaborate, expected length of stay *≥* 5 days, absence of clinical instability and severe dementia (Global Deterioration Scale 7) or other end-stage disease. Patients will be randomly assigned to a control group (CG) or any of the three intervention groups (IG): IVR, ME, or IVR + ME. The IVR group will watch ad-hoc videos showing Spanish regional landscapes and villages, approximately 4 min per day for three consecutive days. The ME group will undergo aerobic and strength exercise for progressive training of the upper and lower limbs. The IVR + ME group will do both cognitive and physical intervention. The primary outcomes will be cognitive and physical measures at discharge. Mood, quality of life, isometric strength, and acceptance of IVR will be also assessed.

**Discussion:**

This project has the potential to enhance physical and psychological well-being of patients with severe functional dependency hospitalized for acute conditions, using technology. Virtual reality is expected to be favourably perceived by hospitalized older adults. This intervention represents a novelty in the geriatric patients’ care, comprising IVR and/or ME dispensed within the patient’s room, and including patients who are commonly excluded from research clinical trials.

**Trial registration:**

This study was approved by the Navarra Clinical Research Ethics Committee on May 17th, 2023 (PI_2023/60). The trial is registered at ClinicalTrials.gov, registration number NCT06340282, 24th May 2024.

**Supplementary Information:**

The online version contains supplementary material available at 10.1186/s12877-024-05516-x.

## Introduction

The global trend of population aging has been steadily increasing over the past several decades, and it is expected to exponentially grow in the next years [1]. By 2050, the individuals aged 65 years and older will reach 2.1 billion, doubling from 2015 [2]. Additionally, projections for the oldest old population indicate that this group of individuals could triple by 2050, reaching a total of 434 million [3]. Together with the population ageing, the prevalence of frailty and multimorbidity has been progressively increasing [4, 5]. Frailty is a geriatric syndrome characterized by an excessive vulnerability of the individual to endogenous and exogenous stressors [1], associated with poorer health outcomes, such as increased risk of death, disability, reduced quality of life, increased likelihood of adverse drug events [4], and higher risk of hospitalization [6].

Almost 30% of the older adults experience functional decline and loss of autonomy in the activities of daily living (ADL) during hospitalization [7, 8]. Hospital-associated functional decline is also associated to increased risk of delirium, reduced functional mobility [9], and reduced quality of life upon discharge [7, 10, 11], as well as loneliness, increased risk of depression, anxiety, and apathy [10–12].

The functional decline in geriatric inpatients has been recently addressed through the implementation of multicomponent physical training programs [10, 13] or technology-based gamification interventions [14]. However, there is limited knowledge about implementation of interventions in older inpatients with severe dependency (Barthel Index < 60 points [15] ), since these patients are usually systematically excluded from clinical trials due to their functional limitations. Therefore, it is essential to develop efficient, motivating, standardized, and widely accessible interventions for these older adults [16], combining cognitive stimulation and physical exercise in order to prevent both cognitive and functional decline [17, 18].

Nowadays, technological solutions are becoming more affordable and accessible, thus representing a resource on which we may count on. Virtual reality (VR) is a promising intervention technique that has already been used with children [19, 20], adolescents, and adults [20, 21] in various conditions, including hospital settings [22]. Commercial devices such as Wii^®^ (Nintendo Company, Ltd., Kyoto, Japan), Microsoft Kinect^®^ (Microsoft Corporation, Redmond, Washington, USA), and interactive video dance gaming have been also used as interventions among older people, with positive opinions and effects on the physical and cognitive domains [23–27]. However, there are potential limiting factors, such as the vision/hearing impairments, and the digital gap. Nevertheless, glasses and audiphones are compatible with VR devices [12, 28] and the technological gap has been narrowing between younger people and older adults [29].

VR can be classified into three categories based on the degree of immersion [30, 31], having distinct characteristics. The first category includes non-immersive systems, such as desktop computers; the second are semi-immersive projection systems, such as large screen monitors; and thirdly, full immersive systems, such as Head-Mounted Displays (HMD). The latter provides users with a complete view of a situation and a higher level of immersion. VR has been showing promising results in cognitive and psychosocial fields, including reducing depression, anxiety, apathy, and increasing cognition, affect, quality of life, and visuospatial function in non-hospitalized older adults [12, 17, 23, 24]. Additionally, VR is an affordable, feasible, and safe tool without side effects, which can encourage older adults to break their daily routine [12, 23, 24, 32].

Despite a recent increase in similar studies in the geriatric population, there is still a lack of research in hospitalized older adults with functional and cognitive impairment. In this population, VR may offer an alternative strategy that could shift treatment of patients with impairments from a traditional management to innovative solutions.

The physical domain of geriatric inpatients has been studied extensively in various settings over many years [10, 33]. However, it is challenging to replicate resistance protocols with aerobic exercises in patients with severe functional dependency (Barthel Index < 60 points [15] ). To address this issue, a specific cycloergometer designed for frail patients offers three types of training: passive, active-assisted, and active exercises (MOTOmed^®^ model MUVI). This tool may help geriatric patients increasing not only mobility [34, 35] and resistance, but also cognitive domain, favouring perfusion in both muscle and brain tissues [13, 33]. However, it remains unclear whether this type of exercise is feasible for geriatric inpatients and what specific effects might have on them.

The impact and consequences of immersive VR (IVR) and/or multicomponent exercises (ME) on hospitalized older individuals with severe functional dependency have yet to be explored.

This study protocol aims to describe a randomized controlled clinical trial with allocation to parallel groups (1:1:1:1), which will examine the effect of IVR and/or ME on cognitive and functional domains, in addition to mood, satisfaction, and technology acceptability, compared to the usual care delivered in hospitalized older adults with severe functional dependency.

## Methods

### Study design

The study will be conducted in the Acute Geriatric Unit (AGU) of a tertiary hospital in Pamplona, Navarra, Spain (Hospital Universitario de Navarra, HUN). The AGU is a 50-bed ward, and its staff is composed of 16 geriatricians, nurses, and physiotherapists.

The protocol satisfies the standard items for clinical trials according to the SPIRIT 2013 statement [36] (see checklist Additional file 1), and follows the CONSORT 2010 statement [37]. As for the data monitoring, a formal committee is not requested due to the type of study.

### Eligibility criteria

Medical inpatients admitted to the AGU of HNU between October 2023 and March 2025 will be evaluated for the eligibility criteria of the study from geriatricians (Table 1). Admissions in the AGU are mainly derived from the Emergency Department, with pulmonary and infectious diseases being the main causes of admissions.

**Table 1:** Eligibility criteria

Inclusion	Exclusion
Age ≥ 75 years	Barthel Index score ≥ 60 at admission
Stability in cardiopulmonary, respiratory, and neurological systems (based on vital parameters and absence of delirium)	Refusal to sign the informed consent by the patient/legal guardian or inability to obtain it
Expected length of stay ≥ 5 days	Life expectancy minor than three months
Being able to communicate and collaborate with the research team	End-stage disease
	Severe level of major neurocognitive disorder (GDS 7)

### Inclusion criteria

- ◦ People aged ≥ 75 years admitted to the AGU of HUN.
- ◦ Stability in cardiopulmonary, respiratory, and neurological systems (based on vital parameters and absence of delirium).
- ◦ Expected hospital length of stay ≥ 5 days.
- ◦ Ability to communicate and collaborate with the research team.

### Exclusion criteria

- ◦ Barthel Index score ≥ 60 at admission.
- ◦ Refusal to sign the informed consent by the patient/main caregiver/legal guardian or inability to obtain it.
- ◦ Life expectancy minor than three months.
- ◦ End-stage disease.
- ◦ Severe level of major neurocognitive disorder (Global Deterioration Scale, GDS Fast Reisberg 7).

The participants, legal tutor, or their acquaintances will provide their written informed consent to participate in this study and for the obtainment of biological samples (see Additional files 2,3).

### Description of interventions and usual care

**Fig. 1:**
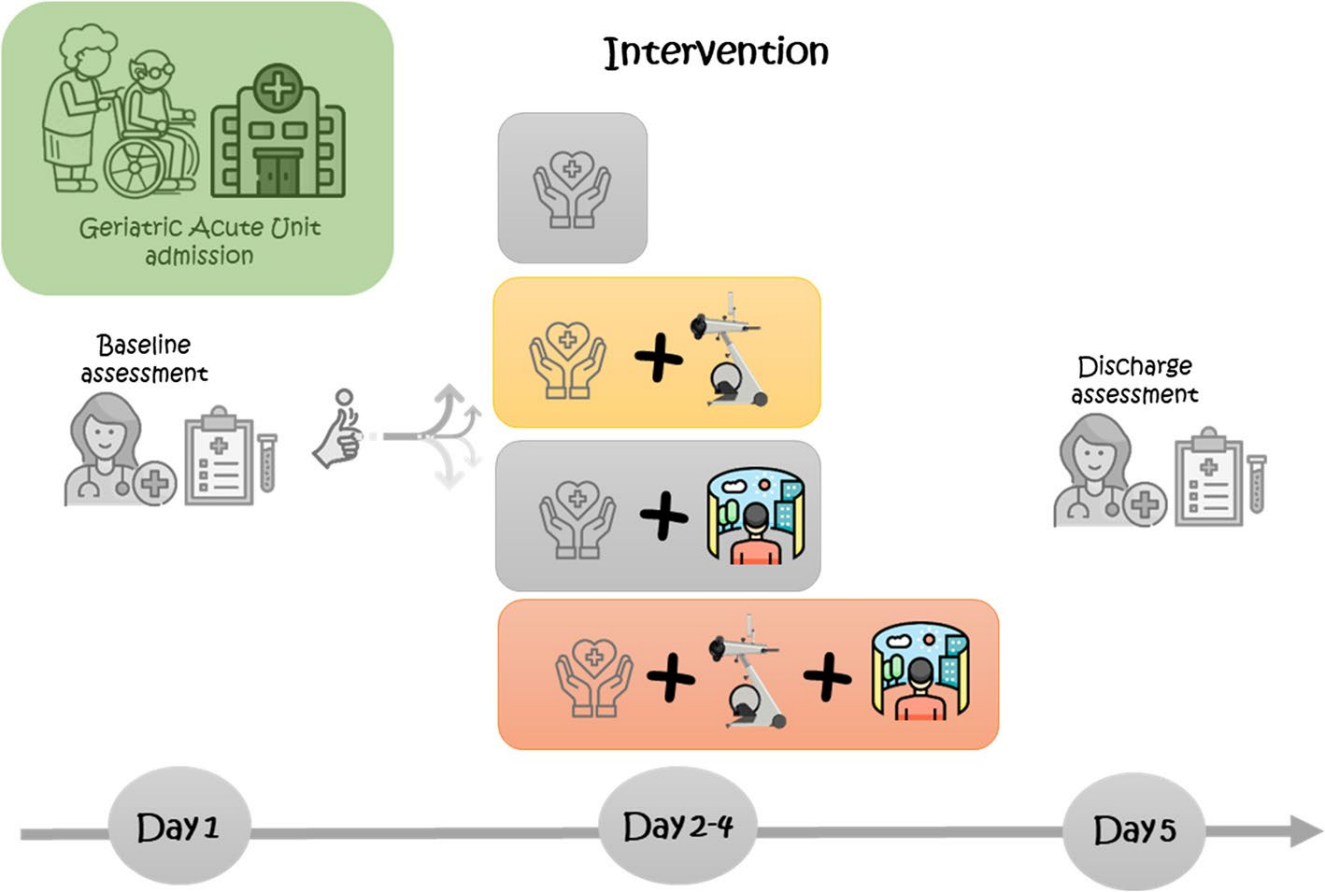
Intervention timeline

Patients will be randomly assigned to the following described control group (CG) or to any of the following intervention groups (IG). Participants or their relatives will be able to discontinue the study for any reason at any time. Geriatricians of the AGU will offer physiotherapy whenever considered necessary, regardless the group assignment of patients, since restoring functional movements and reducing the physical decline during hospitalization is part of the usual care of AGU of HUN. Figure 1.

### IVR stimulation (IVR)

The intervention will be programmed once a day (preferably in the morning) for three consecutive days (including weekends), lasting approximately 4 min. Two ad-Hoc 3D videos, produced by Nautilus Enterprise (Pamplona, Navarre) will be available for this purpose. On the 1st and 2nd day, one of the videos is reproduced through the HMD. On the 3rd day of the intervention, the patient will decide which video to watch.

The first video has two different natural settings: (a) Irati Forest; (b) Bardena desert. The second video is a simulation of visiting villages and cities of Navarre, going by car through landscapes, squares with people walking, iconic buildings, and ending with a drone view of a small village. Both the 3D videos were developed in the Navarre region, with the aim of making the stimulation familiar to most of the patients. The videos were recorded using 6 cameras (Insta Pro 2, Shenzhen, Guangdong, China); afterwards, Nautilus Enterprise performed image stitching, editing, colorization, sound, and rendering of the video. The final export was performed at a quality of 5760 × 2880, with a data transfer rate of 80 MB/s. A Matrice drone (Shenzhen, Guangdong, China) was used for the drone video. Regarding the vehicle used for some parts of the videos, considering carbon footprint, an electric car was used, trying to reduce to the minimum the ambient impact.

**Fig. 2:**
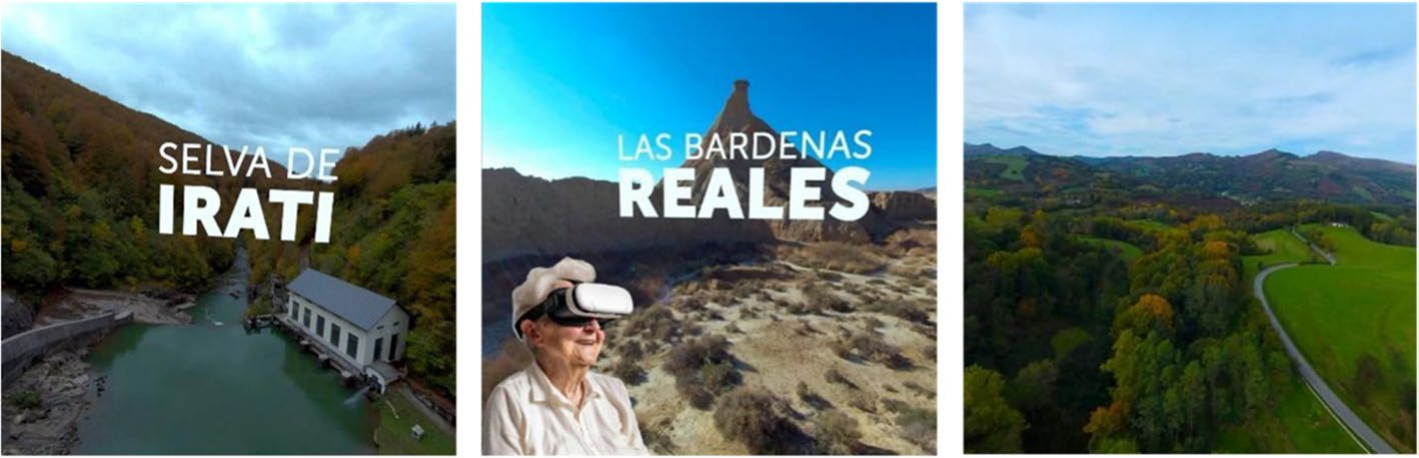
Images from the VR glasses display

The research staff will continuously supervise the intervention sessions. For safety reasons, patients will seat in armchair during the stimulation, and he/she will never stand up. A daily record will be documented. The participants and their family members or caregivers will be familiarized with the IVR and the procedure before starting the intervention. To make it easier and more feasible the VR session, it will be performed in own patients’ hospital room. Figure 2.

### Multicomponent exercise Group (ME)

Patients will undergo progressive training of the upper and lower extremities, as summarized in the exercise protocol (Table 2):

- Hand grip strengthening, through ball squeezes (2 sets of 10 repetitions);
- Seated row exercise with resistance bands (for dorsal, biceps and core muscles), (2 set of 10 repetitions first day and 3 of 10 repetitions in the second and third days);
- Chair rises / Assisted half squats (3 sets of 5 repetitions). If this is not possible, there will be the same repetitions of knee extension with 0.5/1/2 kg, depending on the patient strength (2 set of 12 repetitions first day and 3 of 10 repetitions in the second and third days);
- Cycle ergometer exercise. The intensity will be measured using the Borg Scale [38], after patients will be familiarized with this scale.

The research staff will continuously supervise the intervention sessions. A daily record was documented. Participants and their family members or caregivers will be familiarized with the cycloergometer and procedure before the start of the intervention.

**Table 2:** Exercise protocol

Exercise	1st Intervention Day	2nd Intervention Day	3rd Intervention Day
Handgrip	2 × 10	2 × 10	2 × 10
Row exercise (resistance band)	2 × 10	3 × 10	3 × 10
Chair raises (assisted half squat)	3 × 5	3 × 5	3 × 5
Cycle ergometer Upper limb	5’ (3–4 Borg)	7’ (3–4 Borg)	7’ (3–4 Borg)
Cycle ergometer Lower limb	2’ (3–4 Borg)	3’ (3–4 Borg)	3’ (3–4 Borg)

### IVR stimulation + Exercise Group (IVR + ME)

This group will receive both the cognitive and physical interventions of the two previous groups.

### Control Group (CG)

Control group will receive the usual care, which focuses on health restoration and pharmacological optimization.

#### Primary outcome measures

- Mini-Mental State Examination (MMSE) [39] (a 30-point questionnaire; scale of 0 [worst] to 30 [best]).
- Isometric handgrip strength: the dominant-hand strength will be measured using a hand dynamometer (Takei Scientific Instruments Co., Tokyo, Japan). Patients will sit down with the elbow on complete extension and 5–10º of abduction. From this position, they will be asked to squeeze the handle as forcefully as possible for 3” twice. The highest result will be considered for data collection.
- Hierarchical Assessment of Balance and Mobility (HABAM) scale. The daily functional status will be assessed with the HABAM, an instrument that provides a clinical assessment of in-bed mobility, transfers, and ambulation [40]. A value of 0 is equal to the lowest or inability of performance. Changes in performances can be recorded with patient progress.

#### Secondary outcome measures

a Mood: 15-item Spanish Geriatric Version of Geriatric Depression Scale [41] (from 0 [best] to 15 [worst]); Short Form of State Trait Anxiety Inventory adapted to Spanish Geriatric Population [42] (STAI) (13-point questionnaire, from 0 [best] to 13 [worst]);
b Trail Making Test-A (TMT-A): cognitive test, which requires patients to draw sequentially lines connecting 25 encircled numbers distributed on a sheet of paper [43].
c Quality of life: EuroQol 5-D 3-L (Eq. 5D-3-L) questionnaire [44] to measure five dimensions of health status (mobility, self-care, usual activities, pain/discomfort, and anxiety/depression), and with a Visual Analogue Scale to quantify instantaneous perceived self-health from 0 [worst health state imaginable] to 100 [best health state imaginable].
d Questionnaires about the intervention: Simulator Sickness Questionnaire (SSQ) [28] to assess cybersickness or secondary effects of VR (translated and adapted for the Spanish population); System Usability Scale [45] to evaluate the usability of VR (10 questions with answers from 1 [lower] to 5 [higher]. Additionally, ad-hoc tables will be prepared to register adverse events, self-perceived grade of difficulty, satisfaction, enjoyment, and falls while performing the intervention. Heart rate and oxygen saturation will be measured twice during each intervention-day (after watching the video in IVR or IVR + ME groups). Moreover, agitation will be measured using the Richmond Agitation-Sedation Scale [46] (RASS) (punctuation from + 4 [combative] to -5 [deep sedation]).
e Functional performance: Barthel Index over a 100-point scale (from 0 which represents severe functional dependency up to 100, functional independency) [15].
f Delirium will be measured by using the Confusion Assessment Method (CAM) [47], a 5-question-brief test with information that can be provided by the family or nurse team.
g Comorbidities and polypharmacy: Cumulative Illness Rating Scale-Geriatric (CIRS-G) [48] and changes in the number of drugs and psychotropic at discharge.
h Length of hospital stay (days).
i Follow-up will be performed 1 month after discharge by phone call. Institutionalization, readmission and mortality rate will be assessed through clinical history review. Furthermore, medical staff will re-assess polypharmacy (changes in the number of drugs and psychotropic medication compared to baseline assessment).

Completed personal data or other documents containing protected personal health information will be stored in a locked file at the principal investigator’s office. The data will be entered into an electronic de-identified database authorized by a study team member and checked for completeness and accuracy. Access to data using identifiers is strictly restricted by authorized study team members and authorities. Electronic data will be securely stored on a server regulated by a local research institute (NAVARRABIOMED). Any identifiable data will be maintained until consent is revoked. The times at which different variables will be measured are listed in Table 3 (outcome variables will be assessed by dedicated medical staff).

**Table 3:**
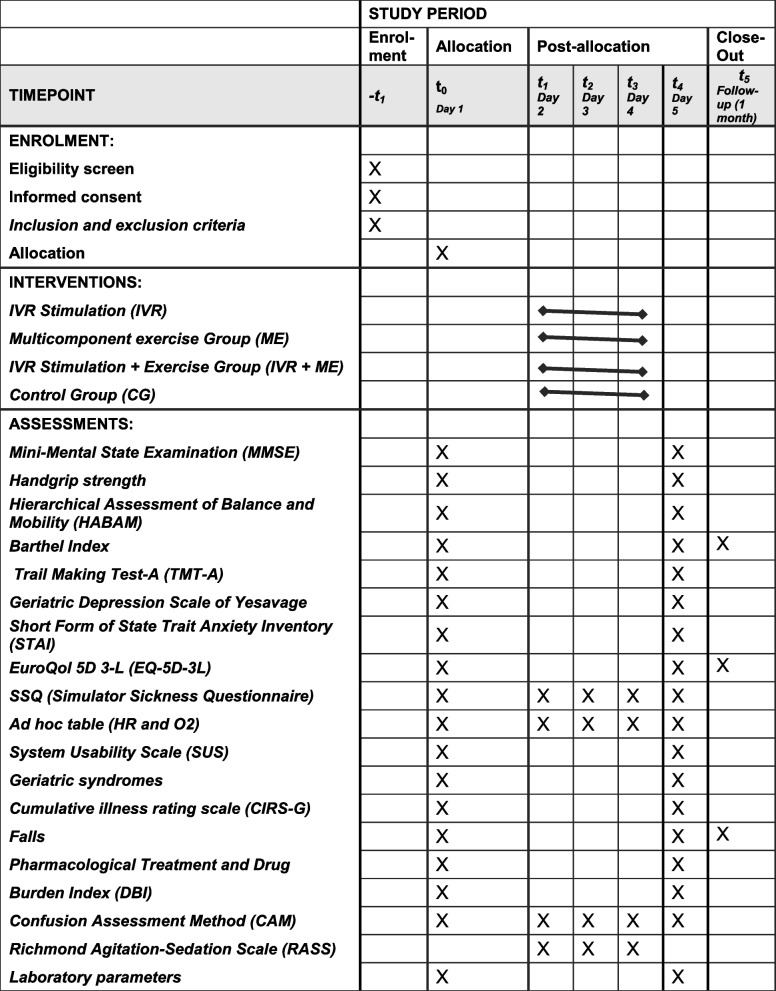
SPIRIT figure: schedule of enrolment, interventions, and assessments

Plans to promote participant retention and complete follow-up include email or text message reminders about appointments, participant empowerment, and resource facilitation.

#### Sample size

To detect a cognitive improvement, measured by the MMSE as a difference greater than 3 points at discharge between groups (bilateral α = 0.05, power = 0.90), 45 patients should be enrolled in each group. Assuming 15% losses, the final goal of each group is 53 patients, and consequently, a total sample size of 212 subjects.

#### Randomization and blinding

All patients hospitalized in the AGU will be screened for the eligibility criteria. The ones who meet the eligibility criteria will be randomly assigned to one of the IG or to the CG within the first 48 h of admission, following a simple randomization procedure in a 1:1:1:1 ratio without restriction. Randomization will be performed using the randomizer software (https://randomizer.org/) with a random permuted block design with block sizes.

**Fig. 3:**
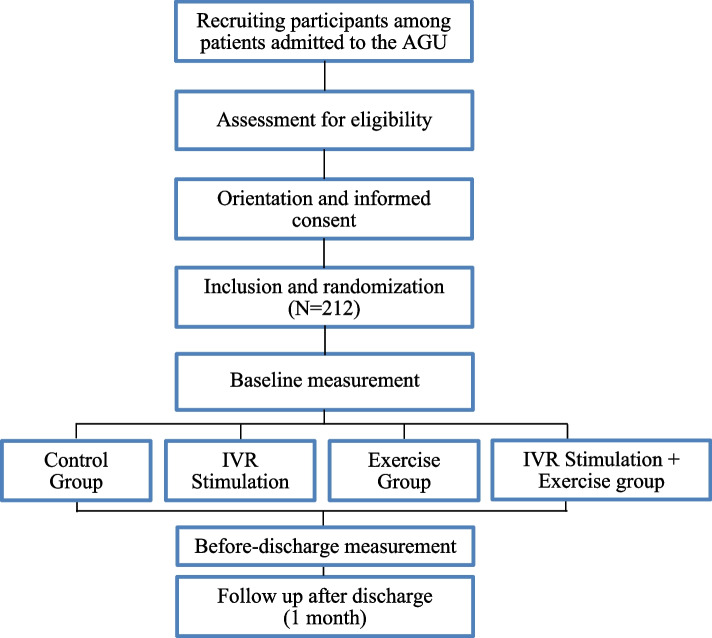
Flow diagram

Participants will be explicitly informed and reminded not to discuss their randomization assignment with the assessment staff. The assessment staff will be blinded to the participants’ group and the main study design. It is not possible to mask the group assignment to the staff involved in the IG training. Patients and caregivers/legal guardians (in case the patient has cognitive impairment) will be informed of their random inclusion in one group but will not be informed on the group in which they are. Figure 3.

#### Statistics

Initially, a descriptive analysis will be carried out in the total sample and in the groups. As for continuous variables, the statistics of central tendency and dispersion will be calculated according to the variable distribution (mean and standard deviation or median and interquartile range), whereas frequencies and percentages will be considered for qualitative variables, with respective confidence intervals in the overall prevalence. The baseline variables will be compared between the treatment and control groups using the Student’s t-test or Mann–Whitney U test for quantitative variables, and the Chi-square test or Fisher’s test for qualitative variables. The effect of the intervention on the different outcome variables will be analysed using ANCOVA models or linear mixed models for quantitative variables; logistic models, differences in rates or differences in proportions will be also performed for falls, mortality, and institutionalization. If any independent variable presented relevant baseline differences, a sensitivity analysis will be performed, adjusting for these variables. No missing data imputations are planned. The analysis follows the intention-to-treat principle, but per-protocol analysis will also be performed as a sensitivity analysis. To determine statistical significance, a level of 0.05 will be established. The data will be analysed with SPSS 25.0 and R software v.4.0.

#### Potential sources of bias

There are potential sources of bias in our study that may include selection bias due to the criteria for participant inclusion and performance bias related to the intervention delivery. To mitigate these biases, we will employ randomization for participant allocation and ensure that the intervention is administered by trained personnel who remain blind to group assignments. Additionally, we will conduct regular monitoring to ensure adherence to the protocol and minimize any deviations.

#### Strengths and limitations

This study presents several strengths, notably the innovative application of immersive virtual reality for cognitive stimulation, coupled with a comprehensive multicomponent physical exercise regimen. This dual approach specifically targets a vulnerable population, potentially enhancing both cognitive and functional outcomes. However, there are limitations to consider. The generalizability of our findings may be constrained by the specific context of a tertiary hospital setting, which may not reflect broader community settings. Additionally, individual variability in responses to the interventions could impact the overall effectiveness.

## Discussion

Frailty and cognitive impairment are common geriatric syndromes in hospitalized older individuals, especially among those with severe functional dependency. To date, no randomized clinical trial has been involving these patients in a cognitive and functional intervention - dispensed through IVR and/or ME -, with the final aim of reducing hospital-associated cognitive and functional decline. Although our research group has been working with older inpatients for over a decade using multicomponent and VIVIFRAIL prescriptions, we previously focused only on autonomous patients. An innovative aspect of this study is to target those patients who are commonly unable to follow a traditional training, including those affected by dementia and functional impairment. Another remarkable novelty of this study is the strict cooperation of an interdisciplinary team, (including physiotherapists, geriatricians, physical education doctors, nurses, engineers), to provide comprehensive care and to reduce the inter-generational digital gap.

If our hypothesis is accurate, this project has the potential to enhance physical and psychological well-being of patients with severe functional dependency hospitalized for acute conditions, through virtual reality and physical exercise. Furthermore, this trial will inform upon eventual adverse events that may occur during the use of IVR or while performing ME, as well as upon the usability and acceptability of technology in this population. This trial represents a novelty in the geriatric patients’ care and may pave the way for other innovative interventions in the future.

### Status of trial

The trial is registered at ClinicalTrials.gov, number of registration NCT06340282, 24th May 2024. The study will be implemented and reported in accordance with the Standard Protocol Items: Recommendations for Interventional Trials (SPIRIT) Guidelines.

## Supplementary Information

Additional file 1.

Additional file 2.

Additional file 3.

## Data Availability

No datasets were generated or analysed during the current study.

## Abbreviations

ADL: activities of daily living
AGU: Acute Geriatric Unit
CAM: Confusion Assessment Method
CG: control group
CIRS-G: Cumulative Illness Rating Scale-Geriatric
GDS: Global Deterioration Scale
EQ5D-3-L: EuroQol 5-D 3-L
HABAM: Hierarchical Assessment of Balance and Mobility
HMD: Head-Mounted Displays
HUN: Hospital Universitario de Navarra
IG: intervention groups
IVR: Immersive virtual reality
ME: Multicomponent exercise
MMSE: Mini-Mental State Examination
RASS: Richmond Agitation-Sedation Scale
SSQ: Simulator Sickness Questionnaire
STAI: Short Form of State Trait Anxiety Inventory adapted to Spanish Geriatric Population
SUS: System Usability Scale
TMT-A: Trail Making Test-A
