# Supplementary material for: Effects of immersive virtual reality stimulation and/or multicomponent physical exercise on cognitive and functional performance in hospitalized older patients with severe functional dependency: study protocol for a randomized clinical trial
Source: BMC Geriatr. 2024 Nov 8;24:924. doi: 10.1186/s12877-024-05516-x (PMC11545771; doi:10.1186/s12877-024-05516-x)
Supplement: Supplementary file 2 — Additional file 2. [file 12877_2024_5516_MOESM2_ESM.pdf]

# PREVENCIÓN DEL DETERIORO COGNITIVO A CORTO Y MEDIO PLAZO MEDIANTE UNA INTERVENCIÓN CON REALIDAD VIRTUAL EN ADULTOS MAYORES HOSPITALIZADOS: ENSAYO CLÍNICO ALEATORIZADO

# HOJA DE INFORMACIÓN AL PACIENTE

**Estimado Sr/Sra:**

Se le ha invitado a participar en un estudio cuyo objetivo es analizar si una intervención mediante tecnología de Realidad Virtual mejora la capacidad cognitiva a corto y medio.

Este documento es un formulario de consentimiento informado y le ofrece información más detallada sobre el estudio. Antes de que usted otorgue su consentimiento para participar en el estudio es importante que comprenda el objeto del mismo. Una vez haya tenido ocasión de leer detenidamente este formulario y comentar con el médico las dudas que pueda tener, deberá firmar el formulario si deseaparticipar.

## 1 NATURALEZA Y OBJETO DEL ESTUDIO

Habitualmente los pacientes ancianos frágiles sufren deterioro en los aspectos funcionales y cognitivos durante la hospitalización, lo cual condiciona un deterioro notable de su calidad de vida y de la capacidad de mantenerse independiente. Estos aspectos suelen generar una necesidad de cuidados por parte de familiares u otras personas e incluso el ingreso en una residencia.

Mediante el uso de Realidad Virtual Inmersiva, en este estudio pretendemos mejorar la calidad de vida y los problemas cognitivos en ancianos frágiles.

Mediante este **ensayo clínico aleatorizado** se valorará la efectividad y seguridad del uso de Realidad Virtual en pacientes geriátricos hospitalizados. La participación en un ensayo es una decisión voluntaria y personal. En el caso de no querer participar o bien de querer abandonar el estudio, la calidad de la asistencia que recibirá no se verá afectada y se seguirán los protocolos habituales. Si decide participar, se le entregará la Hoja de Información al paciente y el Consentimiento Informado para que firme todas las hojas de ambos documentos.

La intervención consiste en ver durante 3 días, un video por día. Son videos de realidad virtual Inmersiva con duración de 4-8 minutos cada video. Son videos grabados en 360 grados en que los/as pacientes vivenciarán la experiencia/sensación de que están en la naturaleza y/o en el medio rural o urbano (dependiendo del video) de localidades de Navarra.

Su negativa a participar en el mismo no repercutirá en su asistencia médica presente o futura. Si usted desea participar, se obtendrá dos muestras de sangre durante la hospitalización (al empezar el estudio y al finalizarlo). Además, recibirá una visita del investigador para recabar una serie de datos de su historia clínica y realizar varios cuestionarios durante unos 30 minutos aproximadamente. Accederemos a los datos de su historia clínica para obtención de datos relacionados con el presente proyecto: visitas a urgencias, reingresos, fármacos. Durante los siguientes 10 años monitorizaremos datos relacionados con su salud accediendo a la Historia Clínica Informatizada de Servicio Navarro de Salud.

## ¿POR QUÉ LE INVITAMOS A USTED A PARTICIPAR EN NUESTRO ESTUDIO?

Le invitamos a participar en este estudio debido a que usted cumple con los criterios necesarios para la inclusión en el mismo. Dada la naturaleza del estudio, mediante la aceptación de compartir con los investigadores datos sobre su salud tomados de diferentes fuentes como son las consultas y la historia clínica informatizada de nuestrohospital, usted habrá podido ayudar a la investigación en el ámbito hospitalario.

## ACLARACIONES

El tiempo aproximado de entrevista clínica será en torno a 30 minutos, en el periodo que no interfiera con su tratamiento. Se realizarán una serie de cuestionarios y test que valorarán su estado de salud, social y calidad de vida para poder realizar posteriormente un análisis más detallado de los resultados, y la aceptación de compartir con los investigadores datos sobre su salud tomados de diferentes fuentes como son las consultas y la historia clínica informatizada de nuestro hospital. Estos cuestionarios se realizarán en cada consulta que acuda a ser evaluada por el equipo clínico y de investigación, y según sea primera consulta o consulta de seguimiento, se recopilará la información perteneciente a cada cuadernillo de recogida de datos. Se tomarán muestras sanguíneas 10-20 mL previo ayuno de 10-12h en las valoraciones inicial y final para su posterior análisis. Estas muestras coincidirán siempre que posible con las muestras sanguíneas rutinarias del ingreso hospitalario.

Si finalmente desea involucrarse en el estudio es necesario que firme el consentimiento informado. El hecho de que inicialmente acepte participar, no significa que no pueda retirarse de la investigación en el momento que usted desee y en ningún caso se le exigirán explicaciones.

La firma del consentimiento informado autoriza a los investigadores principales a recabar datos sobre su salud de diferentes fuentes de información como son las consultas y la historia clínica informatizada de nuestro hospital. Toda esta información será mantenida en la más estricta confidencialidad por el investigador. Cualquier dato que se recoja en el estudio será confidencial.

## PROTECCIÓN DE DATOS Y CONFIDENCIALIDAD

Este proyecto respeta los principios fundamentales establecidos en la Declaración de Helsinki (Asamblea Médica Mundial), en el Convenio del Consejo de Europa relativoa los derechos humanos y la biomedicina, en la Declaración Universal de la UNESCO sobre el genoma humano y los derechos humanos, y cumple los requisitos establecidos en la legislación española en el ámbito de la investigación biomédica, la protección de datos de carácter personal y la bioética. Los investigadores se comprometen al cumplimiento del Reglamento Europeo de Protección de Datos (RGPD UE 2016/976) y Ley Orgánica 3/2018, del 5 de diciembre, de Protección de Datos Personales y garantíade los derechos digitales. Los datos recogidos para el estudio estarán identificados mediante un código, de manera que no incluya información que pueda identificarle. Elacceso a su información personal identificada quedará restringido al equipo clínico delestudio/colaboradores, autoridades sanitarias, al Comité de Ética de la Investigación y personal autorizado por los investigadores, cuando lo precisen para comprobar los datos y procedimientos del estudio, pero siempre manteniendo la confidencialidad delos mismos de acuerdo a la legislación vigente. Por lo tanto, su identidad no será revelada a persona alguna salvo excepciones en caso de urgencia médica o requerimiento legal. El tratamiento, la comunicación y la cesión de los datos de carácter
personal de todos los participantes se ajustarán a lo dispuesto en esta ley.

Los datos obtenidos se guardarán en una base de datos de acceso restringido a los investigadores que participan en el estudio, ubicada en un despacho del Servicio de Geriatría cuyos responsables custodiarán. El consentimiento podrá ser denegado y/o revocado por su representante legal o persona vinculada en el caso de personas incapacitadas legalmente. Los datos serán conservados durante 10 años y posteriormente eliminados.

## RIESGOS POTENCIALES

El programa de Realidad Virtual al que le vamos a exponer está adaptado a su situación clínica y supervisado por profesionales formados para realizarlo. Los profesionales médicos estarán al tanto del desarrollo del mismo y ante cualquier riesgo se interrumpirá y reevaluará.

## BENEFICIOS

Los resultados de este estudio permitirán continuar desarrollando estrategias óptimas para la optimización del periodo de hospitalización en personas mayores, mejorando así el proceso asistencial. Se espera manutención o mejoría en las capacidades cognitivas, y en la calidad de vida; bien cómo disminución de fármacos en caso de pacientes hiperactivos en comparación a los que no reciben una intervención de ambiental como la que proponemos.

CUALQUIER DUDA QUE LE SURJA ANTES DE TOMAR UNA DECISIÓN PUEDE ACLARARLACON SU MÉDICO.

**¿CÓMO PUEDO ESTABLECER CONTACTO SI NECESITO OBTENER MÁS INFORMACIÓN O AYUDA?**

Mediante la firma de este formulario, usted asiente que ha estado informado de las características del estudio, ha entendido la información y el/la doctor/a ha clarificado todas sus dudas. Puede pedir más información o solucionar cualquier duda sobre su participación en este o cualquier momento a lo largo del estudio contactando con el Dr. Martínez Velilla del Hospital Universitario de Navarra.

Nombre del Investigador Principal:
Dr. Nicolás Martínez Velilla

NAVARRABIOMED-FUNDACIÓN MIGUEL SERVET
C/ Irunlarrea nº 3, 31008, Pamplona (Navarra)
Unidad de Investigación en Geriatría: https://www.navarrabiomed.es/es/actividad-cientifica/grupos-de-investigacion/unidad-de-investigaci%C3%B3n-en-geriatr%C3%ADa

# PREVENCIÓN DEL DETERIORO COGNITIVO A CORTO Y MEDIO PLAZO MEDIANTE UNA INTERVENCIÓN CON REALIDAD VIRTUAL EN ADULTOS MAYORES HOSPITALIZADOS: ENSAYO CLÍNICO ALEATORIZADO

## DOCUMENTO DE CONSENTIMIENTO INFORMADO PARA INCLUSIÓN EN EL ESTUDIO

**DECLARO QUE:**

1. He leído la "Hoja de información al paciente" y entiendo los objetivos del estudio.
2. He sido informado y entiendo que los investigadores principales podrán utilizar información de carácter personal recogida en la historia clínica informatizada para confirmar mi idoneidad y completar los datos necesarios para poder formar parte de la Base de Datos del ensayo clínico.
3. Entiendo que mi participación es voluntaria y que tengo el derecho a abandonar el estudio en el momento que lo desee una vez iniciado.
4. He podido aclarar todas mis dudas respecto a los objetivos del estudio.

**Nombre y apellidos del interesado/a:**

**DNI:**

**Firma del interesado/a:**

En Pamplona, a____de______________de 20____.

**Autorización por representación**. -El consentimiento podrá ser otorgado por su representante legal o persona vinculada por razón familiar o, de hecho, en caso de voluntad del/la interesado/a o incapacidad del mismo, con indicación del carácter con que interviene (representante legal, familiar o allegado).

**Nombre y dos apellidos:**
**DNI:**

En calidad de________________________________________________otorgo la autorización para participar en el proyecto de Investigación.

**Firma:**

En Pamplona, a____de______________de 20____.

---

**DENEGACIÓN / REVOCACIÓN DE CONSENTIMIENTO**

Después de ser informado de los fundamentos y objetivos del presente registro, manifiesto de manera libre **mi DENEGACIÓN / REVOCACIÓN DE CONSENTIMIENTO** para mi participaciónen el mismo.

**Nombre y apellidos del interesado/a:**

**DNI:**

**Firma del interesado/a:**

En Pamplona, a____de______________de 20____.

**Denegación/revocación por representación.** El consentimiento podrá ser denegado y/o revocado por su representante legal o persona vinculada por razón familiar o, de hecho, en caso de voluntad del/la paciente o incapacidad del paciente, con indicación del carácter con que se interviene (representante legal, familiar o allegado).

En calidad de_______________________________________________deniego la autorización para participar en el estudio.

**Firma:**

En Pamplona, a____de______________de 20____.
