# Supplementary material for: Effects of immersive virtual reality stimulation and/or multicomponent physical exercise on cognitive and functional performance in hospitalized older patients with severe functional dependency: study protocol for a randomized clinical trial
Source: BMC Geriatr. 2024 Nov 8;24:924. doi: 10.1186/s12877-024-05516-x (PMC11545771; doi:10.1186/s12877-024-05516-x)
Supplement: Supplementary file 3 — Additional file 3. [file 12877_2024_5516_MOESM3_ESM.docx]

Regarding specimen collection, the blood samples will be collected at the baseline (t0) and before discharge (t4) in the Acute Geriatric Unit of HUN, Pamplona, Spain. Whole blood will be processed (Serum and plasma: EDTA, BCDNA and G) and stored at -80°C, to preserve their integrity and prevent contamination. The voluntary patients will be informed and consented to donate the surplus of samples to Navarrabiomed BIOBANK at the end of the study, in order to carry out other research projects.

The study complies with legal and ethical standards, including protecting participant confidentiality and privacy, and following Institutional Review Board (IRB) guidelines for specimen research involving human subjects.
